# Key factors influencing adoption of an innovation in primary health care: a qualitative study based on implementation theory

**DOI:** 10.1186/1471-2296-11-60

**Published:** 2010-08-23

**Authors:** Siw Carlfjord, Malou Lindberg, Preben Bendtsen, Per Nilsen, Agneta Andersson

**Affiliations:** 1 Department of Medical and Health Sciences, Linköping University, Linköping, Sweden; 2 R&D Department of Local Health Care, County Council of Östergötland, Linköping University, Linköping, Sweden

## Abstract

**Background:**

Bridging the knowledge-to-practice gap in health care is an important issue that has gained interest in recent years. Implementing new methods, guidelines or tools into routine care, however, is a slow and unpredictable process, and the factors that play a role in the change process are not yet fully understood. There is a number of theories concerned with factors predicting successful implementation in various settings, however, this issue is insufficiently studied in primary health care (PHC). The objective of this article was to apply implementation theory to identify key factors influencing the adoption of an innovation being introduced in PHC in Sweden.

**Methods:**

A qualitative study was carried out with staff at six PHC units in Sweden where a computer-based test for lifestyle intervention had been implemented. Two different implementation strategies, implicit or explicit, were used. Sixteen focus group interviews and two individual interviews were performed. In the analysis a theoretical framework based on studies of implementation in health service organizations, was applied to identify key factors influencing adoption.

**Results:**

The theoretical framework proved to be relevant for studies in PHC. Adoption was positively influenced by positive expectations at the unit, perceptions of the innovation being compatible with existing routines and perceived advantages. An explicit implementation strategy and positive opinions on change and innovation were also associated with adoption. Organizational changes and staff shortages coinciding with implementation seemed to be obstacles for the adoption process.

**Conclusion:**

When implementation theory obtained from studies in other areas was applied in PHC it proved to be relevant for this particular setting. Based on our results, factors to be taken into account in the planning of the implementation of a new tool in PHC should include assessment of staff expectations, assessment of the perceived need for the innovation to be implemented, and of its potential compatibility with existing routines. Regarding context, we suggest that implementation concurrent with other major organizational changes should be avoided. The choice of implementation strategy should be given thorough consideration.

## Background

Improvement in quality of health care has gained interest among policy makers in recent years, and bridging the knowledge-to-practice gap is a major concern in many countries. Implementing new methods, guidelines or tools into routine care, however, is a slow and unpredictable process [1], and the factors that play a role in the change process are not yet fully understood [2,3]. Research in the area of implementation emanates from Everett Rogers' studies and theories about diffusion of innovations [4]. Over time, the diffusion paradigm has spread to other fields and areas of specialization, such as knowledge translation, technology transfer and, appearing in the mid-1980s, evidence-based medicine [5].

The diffusion of innovations in health service organisations was studied by Greenhalgh *et al*. [6] in a systematic literature review. They found that the attributes of the innovation, adopter characteristics, contextual factors (inner and outer context) and dissemination efforts are important factors. Regarding technology transfer a study on the adoption of medical devices identified three explanatory variables: subjective expected value of the device, information and learning, and the innovativeness of the adopting unit [7]. The dissemination and implementation of evidence-based guidelines has been studied by Grimshaw *et al*. [8], who conducted a review of publications in the area. They concluded that there is an imperfect evidence base to support decisions about which guideline dissemination and implementation strategies are likely to be efficient under different circumstances. When primary health care (PHC) nurses in Finland were asked about their experiences of guideline implementation, awareness, acceptance and positive consequences of guidelines were factors facilitating implementation, and the adaptation of guidelines to local circumstances was shown to be crucial [9]. A study of the implementation of a new care policy in Swedish health care revealed a more positive view among hospital staff than among PHC staff, and obstacles caused by a frustrating situation were more common in PHC [10].

Theory-based models regarding implementation strategies and built on research findings have been presented. The Promoting Action on Research Implementation in Health Services (PARIHS) framework suggests that implementation success is a function of the nature and type of evidence, the qualities of the context, and the way the process is facilitated [11]. Another model is the knowledge-to-action cycle created by Graham *et al*. [12] stressing both the knowledge creation process and the transference into practice. Greenhalgh *et al*. [6] also provide a conceptual model based on their findings, emphasizing the links between the influencing factors described above.

An important factor in designing implementation strategies for new methods into health care is how to obtain behavioral change among health care providers. Eccles *et al*. [13] advocate the use of behavioral theory in designing implementation strategies. Rogers [4] describes behavioral change as an innovation-decision process that leads either to adoption (i.e. to make full use of an innovation) or rejection (i.e. not to adopt). This process occurs on an organizational level and on an individual level. Similar step-wise models are described in the literature, and can be used to explain change in clinical practice [14]. Not all scientists, however, advocate the use of theory. Bhattacharyya *et al*. [15] state that there is no evidence that theory-based methods are more successful than implementation strategies built on common sense, and Oxman *et al*. [16] argue that no more theory is needed.

As described above, there are a number of factors influencing the adoption of new methods in health care. The PHC setting has its particular difficulties and there is debate on the extent that theory-based implementation strategies should be used. To our knowledge, no previous studies have been conducted in which implementation theory has been used to explain the outcome at the introduction of new methods in Swedish PHC.

The objective of this article was to apply implementation theory to identify key factors influencing the adoption of an innovation being introduced in PHC in Sweden.

## Methods

This is an exploratory study based on qualitative analysis of focus groups involving staff from various PHC teams [17]. Factors that could affect the adoption of a computer-based lifestyle test implemented in PHC were identified.

### Setting

Swedish health care is publicly funded. Hospital care and PHC are provided by the county councils, and PHC has the responsibility to provide preventive services as well as health care to the population. Six PHC units (i.e. health care centres with general practitioners (GPs), nurses and other staff members) volunteered to participate in the study. The size of the units in terms of listed patients is described in Table 1. The age distribution of the patients did not differ substantially between the units. Before implementation, the units were randomized to one of the two implementation strategies described below.

**Table 1:** Size of unit, number of staff members in each staff category participating in interview and, in parenthesis, employed at each unit

Unit	I	II	III	IV	V	VI
Listed patients	13700	10200	6000	13000	9800	7300
GPs	3 (8)	4 (5)	3 (4)	8 (8)	5 (6)	3 (5)
Nurses	5 (19)	5 (11)	2 (6)	4 (13)	4 (12)	7 (15)
Other staff members	4 (13)	2 (5)	1 (1)	4 (9)	1 (3)	3 (11)

### The computer-based tool

The computer-based tool consisted of a touch-screen computer and a printer, placed in a so-called IT kiosk. The computer was equipped with a lifestyle test on alcohol consumption and physical activity. The IT kiosk was placed in a central location in the PHC unit, available for visiting patients, and the staff were encouraged to refer their patients to the computer. Patients who performed the test received a printed test result and tailored advice based on their answers. The computer-based tool test and the scientific basis for using computerized solutions in primary health care has been described previously [18].

### Implementation strategies

Based on the discussion cited in the background to this article [14-16] about the use of theory in implementation studies, two implementation strategies were used to introduce the computer-based tool for lifestyle intervention in PHC: explicit and implicit implementation strategies.

The explicit implementation strategy was based on theories about the innovation-decision process presented by Rogers, including knowledge, persuasion, decision and implementation. Attributes of the innovation, such as trialability and observability, were also taken into account. A change agent from the research team visited the centre to provide an information session (knowledge). A 1-month test period followed, during which all staff members were encouraged to perform the test themselves, and give their opinions about it (persuasion, trialability, observability). At the end of the test period, the change agent visited the centre again; there was a discussion about how the test could be used in the daily routine, and mutual agreement to incorporate it or not as a working method was reached (decision). After the second meeting, the lifestyle test was made available to patients and referral to the test was encouraged (implementation).

The implicit implementation strategy, based not on theory, but rather on what Oxman *et al*. refers to as common sense [16], included an identical information session at the centre by the change agent from the research team. Staff members were given information about the computer-based lifestyle test and about the opportunity to refer their patients to the test. No further dialogue was encouraged, and patient testing could start as soon as the computer with the lifestyle test was installed.

### Groups based on outcome and strategy

To identify key factors influencing adoption, the units were divided into three groups based on outcome and on the implementation strategy used. Outcome (in terms of number of referred patients versus the number of patient visits at the unit) was evaluated after the computer-based test had been in operation for 9 months. Data regarding the number of tests performed and the number of patients referred was obtained from the computer database; data regarding the number of patients visiting each unit was obtained from county council registers. Table 2 shows that unit I had a significantly higher rate of adoption than any of the other units. Thus, the cut-off for adoption was set between unit I and the other five units, i.e. < 43 tests performed per 1000 patients visiting the PHC unit. Unit I had received explicit strategy implementation, thus the groups to be compared were: explicit strategy adopters (Explicit A), explicit strategy non-adopters (Explicit NA) and implicit strategy non-adopters (Implicit NA). In the implicit group there were no adopters.

**Table 2:** Adoption in terms of number of patients referred versus number visiting the units

Unit	Implementation strategy used	Number referred	Patients aged ≥18 years visiting unit	Referred/1000 visits	Risk ratio (compared with unit I)	CI
I	Explicit	262	6075	43	1	
II	Explicit	35	5668	6	0.15	0.11-0.20
III	Explicit	68	2492	27	0.64	0.49-0.82
IV	Implicit	48	4697	10	0.24	0.18-0.33
V	Implicit	57	5499	10	0.24	0.19-0.31
VI	Implicit	38	3676	10	0.24	0.18-0.33

### Data collection

After 9 months of operation of the computer-based test, staff were invited to participate in a focus group interview [17]. Information and an invitation to participate was sent by e-mail to all staff members who had direct contact with patients, and thus could be expected to have had the opportunity to refer patients to the computer. Those answering the mail positively and showing up at the interview session were included in the interviews, and considered a volunteer sampling. The different staff categories were interviewed separately, GPs forming one group, nurses another group and other staff members (nurse assistants, dieticians, welfare officers, occupational therapists) forming a third group at each unit. In total 16 focus group interviews and two individual interviews were conducted, including 67 staff members. Individual interviews were performed when only one member of a certain staff group could participate (other staff members). Group size varied from two to eight members, average four. Number of participants in each group is presented in Table 1. The interviews were conducted in the PHC unit locations, in a room used for staff meetings. One author (SC) served as moderator in all the interviews. The interviews lasted between 35 and 50 minutes, and were observed by an assistant documenting the interaction between the respondents during the interviews, including nonverbal communication. After each session the moderator and the assistant had a brief talk about their impressions. This method for moderator/assistant roles is described by Krueger [19]. Interviews were recorded using a digital recorder, and were transcribed verbatim. Transcription was performed by the moderator in four of the interviews and the others were transcribed by an assistant. After transcription the moderator listened to and read all the transcribed material, and made corrections if necessary.

An interview guide was prepared in advance, based on the authors' knowledge about implementation theories, without pointing out the themes used in the analysis. The guide covered the following areas of interest:

- the overall working situation coinciding with the implementation process

- experiences with the implementation activities

- experiences with using the innovation (in this case the computer-based lifestyle test)

- thoughts about addressing the innovation target area (in this case lifestyle issues)

- openness to innovations at the unit

### Data analysis

Interviews were initially analyzed using manifest content analysis according to Graneheim and Lundman [20]. In directed qualitative content analysis, a theory is applied to deductively compare results [21]. This method, suggested to be useful for analyzing interviews, was found to be relevant for our study [21]. The theory chosen was derived from the conceptual model presented by Greenhalgh *et al*. [6], in which one of the important factors is attributes of the innovation. To further specify these attributes, Rogers' theory about perceived innovation characteristics was applied [4]. This resulted in the following themes: Context, Dissemination, Perceived innovation characteristics and Staff characteristics [6]. The theme Perceived innovation characteristics was divided into the following categories: relative advantage, complexity, trialability, observability, reinvention and compatibility [4]; the categories of the other three themes emerged from the interviews (Table 3).

**Table 3:** Themes, categories and sub-categories used in the analysis

Theme	Category	Sub-category
Context^1^	Working conditions	Work load
		Organizational (or other) change
		Staff situation
	Emotional	Loss of control/frustration
		Hope
		
Dissemination^1^	Decision-making	Expectations
		Involvement
	Activities	Information
		Trial
		Support
	Obstacles	Staff performance
		Routine not established
		
Perceived innovation characteristics^1^	Relative advantage^1^	Advantage
		Disadvantage
	Complexity^1^	
	Trialability^1^	
	Observability^1^	
	Reinvention^1^	
	Compatibility^1^	Not compatible
		Compatible
		
Staff characteristics^1^	Opinions about life style issues in PHC	Importance
		Possibilities
		Obstacles
	Opinions about organizational change	Reluctance to change
		Positive to change

^1^Themes and categories chosen according to the theoretical framework.

For the analysis, the narrative text was read and re-read, and meaning units, that is, words or sentences that are related to each other through content or context [18], were identified throughout the text. The meaning units then were condensed to contain only a few central words, and were labelled with suitable codes. According to the codes, quotes were sorted into categories (or new categories emerged) and themes. This process was conducted by three of the authors, (SC, ML and AA) and codes and categories were discussed until consensus was reached.

### Ethical aspects

According to the Act in Swedish law concerning Ethical Review of Research involving Humans (SFS 2003:460) from the Ministry of Education and Cultural Affairs, the present study required no ethical approval. However, an application for ethical approval was made, and the Ethical Board in Linköping, Sweden, stated in an advisory opinion that the study could be conducted without any further considerations (Ö 16-08). Confidentiality of participants was ensured.

## Results

Interaction between the respondents in the groups showed that they helped each other to relate to the issues included in the discussion. No major disagreements were revealed in the discussions. Table 3 provides an overview of the themes, categories and sub-categories, showing which categories are based on theory and which emerged from the analysis. Tables 4, 5, 6 and 7 provide quotations supporting the results according to the themes, categories and subcategories. Quotes supporting the main findings are also presented in the text.

**Table 4:** Theme: Context

Category	Sub-category	Group		
		
		Explicit strategy: adopters (unit I)	Explicit strategy: non-adopters (units II-III)	Implicit strategy: non-adopters (units IV-VI)
Working conditions	Work load	"We are the same work force ... we follow routines as usual ... some days there's more to do and others less - it depends on how many patients there are and how many are on duty." (Others, unit I)	"Of course things go up and down along with how many patients we have and how things flow ... so it's been, I suppose, normal." (Nurse, unit II)	"It has been, I suppose, a fairly strained situation actually, so much so that there's no time for more than what absolutely must be done, you must make priorities." (Nurse, unit IV)
	Organi-zational change	"Moreover, there are some new things constantly popping up on the computer to be learned." (Nurse, unit I)	"It is neither something that has arisen or disappeared." (Nurse, unit II)	"We had to introduce a new operative computer system while at the same time, reorganize home care." (GP, unit V)
			"We haven't had a manager for several months ... just got a new manager. That is, I guess, the greatest change." (GP, unit III)	"An unbelievable amount has happened here ... doctors in private practice ceased January 1 and we have also gotten a new telephone system." (Others, unit VI)
	Staff situation	"We are basically well manned..." (GP, unit I)	"Many district nurses have been on sick leave lately." (GP, unit III)	"Then we hired in doctors here and were... understaffed ... and new personnel has come in ... the nursing staff was also renewed." (Others, unit VI)
			"Our manager has been sick and absent quite a lot because of that, and that has, of course, been a factor." (GP, unit II)	
Emotional	Loss of control/frustration		"We have gotten by, I suppose." (Nurse, unit III)	"Our wings have been clipped." (Nurse, unit V)
				"It affects the work environment, you could say, there is a higher stress level in some way, and more ... just that you can't feel that you can influence your work environment, either, so to speak, are factors you can't really steer ..." (GP, unit IV)
	Hope			"And we have, I suppose, learned that we can't spend all our energy complaining." (Nurse, unit V)
				"I feel, although, that things have stabilised now - not so much uneasiness amongst the patients." (Nurse, unit VI)

Quotations supporting the results of the different categories, according to groups based on adoption and implementation strategy.

[...], some words left out; ..., hesitation; [ ] author comment.

**Table 5:** Theme: Dissemination

Category	Sub-category	Group		
		
		Explicit strategy: adopters (unit I)	Explicit strategy: non-adopters (units II-III)	Implicit strategy: non-adopters (units IV-VI)
Decision making	Expectations	"One was, in any case, curious and positive." (Others, unit I)	"There was, I guess, no great enthusiasm from any of us, no, you couldn't say that." (GP, unit II)	"We were somewhat sceptical [...] we have much to do anyway and because, perhaps, you should manage your own affairs..." (GP, unit V)
			"We had agreed on that, of course, but ... but we did not, perhaps try hard enough to get as familiar with it as we probably should have." (Nurse, unit III)	"It was a fun or a good thing to bring in." (Others, unit VI)
	Involvement	"There was mostly talk about where to put it, sort of, but not that anyone was opposed to it, as far as I can remember." (GP, unit I)	"Yes, I guess we discussed it, but there was no one who questioned, it was just said that it would come." (Nurse, unit III)	"Yes ... we took it up more as a group whether we were for or against it ... right?" (GP, unit V)
				"Well it seemed it was already decided, when the manager said it, wasn't it at a meeting ...? That it would come ..." (Others, unit IV)
Activities	Information	"Then the manager mentioned it, she was very interested, and keen on bringing it here, then at the personnel meeting everyone got to know when it would arrive." (GP, unit I)	"Yes, there was someone who informed us at a personnel meeting, whoever that was ..." (Others, unit II)	"We went through it properly. All prerequisites for it and how it worked were presented, is what I think." (GP, unit IV)
				"We were all informed and it has worked ever so well." (Nurse assistant, unit V)
	Trial	"I believe practically all personnel were up here testing and comparing results, what we got and ..." (Others, unit I)	"Yes, I believe everyone ... has done it [the test] ..." (Nurse, unit II)	
	Support	"Yes, of course we talk, our S is in the group, she reminds us: Don't forget to refer to the lifestyle computer or write it down. She does ... she reminds us, of course, all the time." (Others, unit I)	"But we do access the statistics, we do." (GP, unit III)	"You need a little push now and again, otherwise..." (Nurse, unit IV)
Obstacles	Routine not established	"And then I forget about it, and then they come for something else, because they are, of course, half sick, or are feverish or, you know, like that ... yes, I forget about it." (Nurse, unit I)	"I forget ... I have had, yes I have told some, yes, I did, but not so many, to be honest." (Others, unit III)	"But it is, you have to remember it when you're sitting there, it has to become a habit, a routine, to refer to it [the computer]." (Nurse, unit IV)
	Staff performance	"Blood pressure patient or diabetic, it is often those you think of first and refer them." (Nurse, unit I)	"It is sort of an ongoing project and ... it's been how it's been ..." (GP, unit III)	"Of course I haven't recommended all my patients to go to this computer, I haven't done that, I must admit it, but we must get tougher about that, all of us." (Nurse, unit VI)
	Timing			"You could, perhaps, call it bad timing" (Nurse, unit V)

Quotations supporting the results of the different categories, according to groups based on adoption and implementation strategy.

[...], some words left out; ..., hesitation; [ ] author comment.

**Table 6:** Theme: Perceived innovation characteristics

Category	Sub-category	Group		
		
		Explicit strategy: adopters (unit I)	Explicit strategy: non-adopters (units II-III)	Implicit strategy: non-adopters (units IV-VI)
Relative advantage	Advantage	"... but that it's for their own good, getting an eye-opener." (GP, unit I)	"Perhaps it will serve as a wake-up call for some patients, you can always hope." (Others, unit III)	"Yes, that it's here, you have to, in general, deal more with these questions ... and then for the patients themselves to ... if they finally do come to the computer, you begin to think about the questions and how one reflects on your situation." (Others, unit VI)
	Dis-advantage	"If it was, thus, some more focus even on smoking [...] miss that part in it, absolutely." (GP, unit I)	"... but sometimes they stand and touch and touch [on the touch screen] and sometimes can't get it to work properly and then it takes time and then they give up." (Nurse, unit II)	"There have even come patients who have thought of doing it and there has been a problem with it, which has happened a few times." (Nurse, unit VI)
Complexity	Complex	"The elderly that don't have computer experience perhaps want you to stand beside and help them out some." (Others, unit I)	"... some who are a little older and not so used to computers, it was like: How do I touch it? How am I to do it?" (Nurse, unit II)	"The elderly don't know what to do, many of them. I mean, they are not used to computers in that way." (GP, unit V)
	Not complex	"People were probably afraid at first that it would take a long time but didn't experience that, just the opposite, that it worked." (GP, unit I)		
Trialability	Trialable	"I thought it was good, I think it's smart to be able to go and test yourself, since you never know - you can't of course send someone to something when you don't know what it is." (GP, unit I)	"And we had it down here then, so that the personnel could test it ... I thought that was good." (Nurse assistant, unit II)	"Perhaps I click on it first - I click and then see how it works." (GP, unit VI)
Observability	Not observable			"I can miss the fact that, I don't know if they go there afterwards, since they usually do that after the visit [...] so you get no feedback on whether they actually were there." (GP, unit IV)
Reinvention	Suggestions for reinvention at own unit	"Actually, for patients coming in to check their blood pressure, you could even begin on the telephone by telling them to take a look at the lifestyle computer before coming in." (Nurse, unit I)	"But then there should, of course, be some information in the waiting room, [...] where the touch screen computer is. Since we don't remember to recommend them to go there." (GP, unit III)	"But I do think, actually, that it would have been best if the reception secretary had said that before you go in to the doctor, please fill this in and take the test results with you to the doctor." (Nurse, unit VI)
Compatibility	Compatible	"As a technical aid ... it is absolutely no bother to refer them to the lifestyle computer, it's not." (Nurse, unit I)		"Couldn't you just say briefly that: You know we have one of those lifestyle computers here, you could try it." (Others, unit IV)
	Not compatible	"It is, moreover, anonymous so it's nothing you can use in clinical work." (GP, unit I)	"I can't find any use for it, because I paint with bigger strokes across the entire spectrum when I speak to my patients ..." (GP, unit II)	"It feels a little bit better to be able to show them to our lifestyle reception where there is someone to talk to them and provide complete answers - all these lifestyle factors ..." (GP, unit V)

Quotations supporting the results of the different categories, according to groups based on adoption and implementation strategy.

[...], some words left out; ..., hesitation; [ ] author comment.

**Table 7:** Theme: Adopter characteristics

Category	Sub-category	Group		
		
		Explicit strategy: adopters (unit I)	Explicit strategy: non-adopters (units II-III)	Implicit strategy: non-adopters (units IV-VI)
Opinions about lifestyle issues in PHC	Importance	"All of society has changed. We are to work preventively now, that's a fact. Practically nothing was said about it 5 or 10 years ago." (Nurse, unit I)	"Yes, but I think people are more aware of how important prevention ís, I think it pervades health care in a completely different way than it did 20 years ago." (GP, unit III) "So - if we could get everyone to exercise, eat sensibly and not smoke, we could just pack up and leave. That, I believe, is the way to a healthier population." (GP, unit II)	"Yes, but it is amongst the most important jobs we have, in fact." (Nurse, unit IV) "Because it is, of course, important for many illnesses or generally speaking it's a question of our lifestyle." (Nurse, unit VI)
	Possibilities	"Yes, we have such a lifestyle team here at the primary health care unit, where we work with different problems. Some work with overweight, some with blood pressure ... I'm to work with tobacco-related problems." (Others, unit I)	"We have, of course, a health coordinator working with lifestyle so that ... doctors refer, of course, to them [...] they take it all." (Others, unit II)	"Everything that brings things into the light, that creates discussion and that gets patients to mention something about it or have seen it in the corridor - I think in some way increases everyone's awareness." (GP, unit V)
	Obstacles	"Healthcare has become so very heavy, I mean, primary health care has become extremely heavy the last 25 years, and I believe still that many had visions ... you lose focus and it just ... but you have to do the most important things ..." (Nurse, unit I)	"We have, I suppose, had a lack of resources ever since we got involved in this ... health project. Actually we don't have ... we were promised more, but nothing came of it." (Nurse, unit III)	"If it is a sleep problem, where it would, perhaps, take less time to write a prescription than to talk about, I don't know ... about exercise ..." (Others, unit IV)
Opinions about innovations, new routines and change	Positive to change	"You want to keep up with the latest news so it is, of course, very good for the primary health care unit." (Others, unit I)	"No, but I have the feeling that openness for testing new ideas is considerably large." (GP, unit III)	"Fantastic "go" in this work group, for everything new ... if something new turns up again, that seems interesting I don't think there would be any difficulties ..." (Nurse, unit V)
				"But this sort of thing that really doesn't demand too much work effort from us ... that is pretty easy to accept ..." (Nurse, unit VI)
	Reluctance to change		"That sort of thing takes both time and energy from us, always something new, new, new to be updated ..." (Others, unit II)	"Yes, we are afraid that changes will cause us even more work ... and that is the reason we have ... a reason that we ..." (GP, unit IV)

Quotations supporting the results of the different categories, according to groups based on adoption and implementation strategy.

[...], some words left out; ..., hesitation; [ ] author comment.

### Context (Table 4)

#### Working conditions

Working situations differed substantially between the units. Explicit A described a heavy but not exceptional work load, continuing changes but no major organizational change, and a staff situation not affected by vacancies or sick leave. Explicit NA had a heavy but normal work load, managers absent due to sick leave or vacancy, staff on sick leave and vacancies, but no major organizational changes. Implicit NA described a period of heavy work load, organizational changes and staff shortages, but had not experienced managers being absent. All units in the implicit group had similar experiences.

We had to introduce a new operative computer system while at the same time, reorganize home care. (GP, unit V)

#### Emotional

The units where staff expressed perceptions at the emotional level were all part of the Implicit NA group. They described feelings of frustration and loss of control due to organizational changes and shortage of staff.

### Dissemination (Table 5)

#### Decision-making

Explicit A staff expressed positive expectations about receiving the computer, and discussed the concept in advance; Explicit NA had no positive expectations, expressed a sense of indifference concerning the computer, a lack of enthusiasm, or were even negative. Staff at Implicit NA expressed skepticism, indifference, or were slightly positive. Staff did not feel involved in the decision to accept the computer at any of the units.

We had agreed on that, of course, but ... but we did not, perhaps try hard enough to get as familiar with it as we probably should have. (Nurse, unit III)

#### Activities

All groups expressed having received enough information about the concept. The opportunity to try the computer (offered at all explicit implementation strategy units) was mentioned by Explicit A and Explicit NA as a positive experience. At Explicit A some staff members did encourage their colleagues; the other two groups mentioned a lack of that kind of support. All groups received and discussed the feedback provided by the change agent.

#### Obstacles

All groups complained about not having established routines for referring, and thus forgetting about it. Explicit A staff expressed disappointment about not referring even more patients to the computer. Explicit NA seemed not to bother about their results. Implicit NA were concerned about their results, and mentioned bad timing and uncertainty about the manager's opinion about the concept as possible explanations. At one Implicit NA unit incorrect information had been circulating that patients should not be referred, only perform the test spontaneously.

### Perceived innovation characteristics (Table 6)

#### Relative advantage

Explicit NA saw few advantages with the computer. The other two groups mentioned a number of perceived advantages with using the innovation including staff becoming aware of the issue, patients becoming aware of their risks, test being performed anonymously, possibility to discuss results, and the test empowering patients to improve lifestyle. Implicit NA, and to some extent Explicit NA had had problems with malfunctioning equipment.

#### Complexity, trialability and observability

Complexity was mentioned in all groups. Staff perceived that elderly patients could find the computer hard to use. Trialability was mentioned not only by the explicit implementation groups (where trying the computer was part of the implementation strategy) but also by the Implicit NA group, where staff had used the opportunity to test the computer when providing the test for patients. Observability was mentioned by Implicit NA, who lacked the opportunity to get feedback on whether a patient being referred to the test actually did perform it.

#### Reinvention

Ideas about reinventing the concept were mentioned by all groups, suggesting that the test should be performed before the consultation, or asking the receptionist to refer patients to the computer. Explicit NAs had ideas about making the test self-distributing, so that staff should not need to refer patients.

#### Compatibility

Regarding compatibility there were substantial differences between the groups. Explicit A saw possibilities with the test, and believed that all patients could benefit from being referred. The test was perceived a valuable tool. Explicit NAs saw very few possibilities with the test, it was not compatible with their routines, and they felt there were better ways to address lifestyle issues. At the Implicit NA units some staff groups saw the possibilities, but overall they had more confidence in and felt more comfortable with referring patients to a lifestyle team at the unit. They also expressed fear about patients perceiving referral as an insult or that the computer would generate more work.

I can't find any use for it, because I paint with bigger strokes across the entire spectrum when I speak to my patients ... (GP, unit II)

### Staff characteristics (Table 7)

#### Opinions about addressing lifestyle issues in PHC

Staff opinions about addressing lifestyle issues in PHC did not differ between the groups. All were concerned about the issue and found it important, but mentioned lack of time and resources. All groups saw possibilities in addressing lifestyle issues and the computer as a tool for that purpose was mentioned by Explicit A and Implicit NA, but not at all by Explicit NA.

#### Opinions about organizational change and innovations

Opinions about new ideas and new working methods were very positive among the Explicit A. Explicit NA were positive, but pointed out that too many changes cause reluctance. Among the Implicit NAs there were opinions from positive to very reluctant to changes. The importance of timing was mentioned and a top-down process was seen as a negative factor.

## Discussion

The four themes suggested in the theoretical framework did serve to cover all the experiences expressed by staff at the PHC units, which shows that the framework is suitable for evaluation of implementation in PHC.

At the adopting unit the explicit implementation strategy had been used, a strategy that has been shown to predict a better implementation outcome if combined with a creative climate at the unit [22]. The Explicit NA group differed from the adopters regarding a number of factors, indicating that these factors may be more strongly linked to adoption than implementation strategy. One of these factors was expectations. Earlier studies on the implementation of clinical guidelines among GPs in PHC state that dissemination must target the perceived needs [23]. Expectations seemed to be more important than staff involvement in the decision process, known to be fundamental in organization development [24]. The intention of the explicit implementation strategy was to involve staff in the decision process, but choice of strategy seems not to have influenced the perception of involvement.

Other important factors were those concerning the innovation characteristics. It was obvious that Explicit NA found the concept incompatible with their way of providing health services, and they saw hardly any relative advantage in using it. The staff seemed to lack a sense of urgency to change, which is consistent with earlier findings in PHC [25]. According to Rogers [4], innovation characteristics explain 49-87% of the variance in rate of adoption of innovations. In a study on the implementation of information systems, Yetton *et al*. [26] found that the contribution of innovation characteristics to implementation success was higher than that of the implementation process. The innovation implemented in this study, the computer-based lifestyle test, was supposed to be perceived as easy to use, possible to try, observable and compatible with existing routines. Computerized solutions in PHC have been evaluated previously and found to be feasible [27]. In this study, the staff in the different groups seem to have diverging perceptions of the lifestyle test characteristics, probably due to factors regarding context or adopter characteristics.

An opinion leader facilitating the implementation was mentioned only by the adopters. In most social systems there are key figures - respected and well-informed professionals who personify the group norms and group culture, and who filter new information and pass it forward [7]. The presence of opinion leaders could have great importance in the implementation process [6]. However, it has been shown that opinion leaders are valuable to support implementation particularly in highly specialized staff groups [28]. The nature of PHC is to address a wide range of health care issues, which could be an explanation for the lack of opinion leaders in this particular setting.

The contextual factors studied were all about the actual inner context - the coinciding working situation. Since all units in the Implicit NA group had had a period of heavy work load, organizational change and staff shortage, it is most likely that this affected their ability to adopt the innovation [29]. The two Explicit NA units both had mangers absent during the study period because of sick-leave or job vacancy, which may have had an impact on adoption because leadership is of great importance for organizational innovation [30,31].

Staff characteristics investigated in this study included opinions about addressing lifestyle issues in primary health care and innovativeness on a group level. The overwhelming awareness about the importance of the issue was not reflected in adoption of the computer-based test. Perceiving it important might lead to an intention to address lifestyle issues, but not necessarily to doing it, since there is a well-documented intention-behavior gap [32]. Positive opinions about change and innovations expressed by the Explicit A probably helped in forming a receptive context, one of the factors that determine innovativeness in health service organizations [16].

The findings from this study might be helpful for policy-makers and for managers and staff in the local setting, aiming to introduce new methods into PHC. A factor not analysed in this study is the contributions of the different professional groups to the results. This will be analyzed further and presented in the future. Another important question that should be assessed in future research is the low overall rates of adoption, and what could be done to encourage the use of the computer-based lifestyle test.

### Limitations and strengths

As in all qualitative research this study is limited regarding its generalizability and relevance to other settings. However, we believe that the knowledge gained from this study could be of importance in planning the implementation of new methods under similar circumstances. The factors assessed in this study could be categorized according to the predefined themes in the theoretical framework. The framework was based on an extensive literature review, and was also consistent with other implementation models described in the literature, which should be considered a strength. Theory, however, contains a number of additional factors that our study was not designed to assess, for example adopter characteristics on an individual level, outer context and networks. However, we believe that the factors assessed do influence implementation outcome in an important way. The fact that size differed substantially between units, and that some of the units experienced organizational changes coinciding with the implementation process might have influenced the results. These are factors that should be considered if the study is repeated.

The main difference between the two strategies was the testing period combined with a decision session provided in the explicit strategy. A more extensive implementation effort might have resulted in higher levels of adoption, but would also have required more financial input. A strength in this study was that a better implementation outcome was obtained despite limited financial resources.

Another strength in this study was that the interviews were conducted with the different staff groups separately, which allowed the individuals to reveal their thoughts without fearing the reactions of staff members in other categories. It is well known that PHC is a hierarchic organization, and a mixed group might have hindered an open discussion.

## Conclusions

When implementation theory obtained from studies in other areas was applied in PHC it proved to be relevant for this particular setting. Based on our results, factors to be taken into account in the planning of the implementation of a new tool in PHC should be assessment of staff expectations, assessment of the perceived need for the innovation to be implemented, and of its potential compatibility with existing routines. Regarding context, we suggest that implementation concurrent with other major organizational changes should be avoided. The choice of implementation strategy should be given thorough consideration.

## Competing interests

One of the co-authors, Preben Bendtsen, holds shares in, and works as a consultant for a company that develops and markets computer-based lifestyle tests similar to the one described in this paper.

## Authors' contributions

SC participated in the design of the study, the data collection, the analysis of data and drafted the manuscript, ML participated in the design of the study, the analysis of data and helped to draft the manuscript, PB and PN contributed to the design of the study and helped to draft the manuscript, AA participated in the design of the study, the analysis of data and helped to draft the manuscript. All authors read and approved the final manuscript.

## Pre-publication history

The pre-publication history for this paper can be accessed here:

http://www.biomedcentral.com/1471-2296/11/60/prepub
